# Antifungal and Cytotoxic Evaluation of Photochemically Synthesized Heparin-Coated Gold and Silver Nanoparticles

**DOI:** 10.3390/molecules25122849

**Published:** 2020-06-19

**Authors:** María del Pilar Rodriguez-Torres, Luis Armando Díaz-Torres, Blanca E. Millán-Chiu, René García-Contreras, Genoveva Hernández-Padrón, Laura Susana Acosta-Torres

**Affiliations:** 1 Laboratorio de Investigación Interdisciplinaria, Área de Nanoestructuras y Biomateriales, Escuela Nacional de Estudios Superiores, Unidad León de la Universidad Nacional Autónoma de México (UNAM), Boulevard UNAM No. 2011, Predio el Saucillo y el Potrero, 37684 León, Guanajuato, Mexico; rgarciac@enes.unam.mx; 2 Centro de Física Aplicada y Tecnología Avanzada, Universidad Nacional Autónoma de México, Blvd. Juriquilla 3001, 76230 Querétaro, Mexico; 3 Centro de Investigaciones en Óptica, A.C., AP 1-948, 37150 León, Guanajuato, Mexico; ditlacio@cio.mx; 4 Departamento de Nanotecnología, Centro de Física Aplicada y Tecnología Avanzada, Universidad Nacional Autónoma de México, Blvd. Juriquilla 3001, 76230 Querétaro, Mexico; genoveva@fata.unam.mx

**Keywords:** heparin, silver nanoparticles, gold nanoparticles, antifungal activity, cytotoxicity, *Candida* spp., human gingival fibroblasts

## Abstract

Heparin-based silver nanoparticles (AgHep-NPs) and gold nanoparticles (AuHep-NPs) were produced by a photochemical method using silver nitrate and chloroauric acid as metal precursors and UV light at 254 nm. UV–Vis spectroscopy graphs showed absorption for AgHep-NPs and AuHep-NPs at 420 nm and 530 nm, respectively. TEM revealed a pseudospherical morphology and a small size, corresponding to 10–25 nm for AgHep-NPs and 1.5–7.5 nm for AuHep-NPs. Their antifungal activity against *Candida albicans*, *Issatchenkia orientalis* (*Candida krusei*), and *Candida parapsilosis* was assessed by the microdilution method. We show that AgHep-NPs were effective in decreasing fungus density, whereas AuHep-NPs were not. Additionally, the viability of human gingival fibroblasts was preserved by both nanoparticle types at a level above 80%, indicating a slight cytotoxicity. These results are potentially useful for applications of the described NPs mainly in dentistry and, to a lesser extent, in other biomedical areas.

## 1. Introduction

Metal nanoparticles, particularly gold and silver ones, have been studied due to their properties such as plasmon resonance and large surface area, among others [1,2,3]. They have an excellent and promising potential use in biomedical applications [4], including bioimaging [5], thermal therapy [6], and drug delivery [7]. They have also been studied for their antimicrobial [8,9,10] and cytotoxic activities [11,12,13], with satisfying results. There are several methods by which metal nanoparticles can be synthesized, involving a wide variety of reactants used as reducing and stabilizing agents, like polymers and surfactants [14,15]. Heparin is a biopolymer with a heterogeneous structure, composed mostly by the repetition of the disaccharide units, glucosamine, and glucuronic acid. It is highly sulfated and negatively charged and is also produced in the body of animals, including humans. Heparin belongs to the glycosaminoglycans family, along with heparan sulfate, hyaluronic acid, and chondroitin sulfate and possesses characteristic properties [16,17]. In clinical medicine, it is generally used as an anticoagulant as well as an anticancer and an anti-inflammatory agent. It is also used as a locking solution in catheters. Additionally, its antimicrobial activity (bacteriostatic) against Gram-positive and Gram-negative pathogens and yeasts (fungistatic) such as *Candida albicans* and planktonic cells has been reported [18]. On the other hand, there are studies which report that heparin does not own such antimicrobial potential [19]. Because of its polyanionic nature arising from carboxylate groups and *O*- and *N*-sulfates [20,21], it is a highly reactive molecule and it has been used in metal nanoparticle synthesis as a capping and reducing agent in wet chemistry approaches [22,23,24,25] and in a photochemical method developed previously by our group [26]. In general, the photochemical method for nanoparticle synthesis has advantages, since it is a clean process with high spatial resolution and versatility (it can be used with a wide variety of materials, e.g., emulsions, surfactants, polymer films, polymers, glasses, and cells) [27]. Additionally, in situ, the generation of reducing agents can be controlled [28]. Heparin-based metal nanoparticles have found interesting applications. For instance, gold nanoparticles capped with heparin have been proposed as a visualization agent for the liver and kidney in computed tomography [29]. Other authors have taken advantage of the binding capabilities of gold nanoparticles to heparin, proposing nanogold-colorimetric sensors to detect the levels of heparin in serum [30]. Furthermore, as for heparin-based silver nanoparticles, Kemp et al. used DAPHP (diaminopyridinylated heparin), a chemically modified heparin, to obtain silver nanoparticles that were assessed for antibacterial activity [31].

*Candida* spp. are pathogenic yeasts that can cause several diseases such as oral candidiasis, urinary tract infections, and candidemia. Among all *Candida* species, *C. albicans* is the most important because of its prevalence, followed by *Candia glabrata*, *Candida tropicalis*, *Candida parapsilosis*, and *Issatchenkia orientalis* [32]. It has been reported that *C. albicans* has heparin-binding motifs that serve as a bridge between cells, facilitating cell–cell adhesion and biofilm formation. Human gingival fibroblasts (HGFs) are important constituents of the gingival tissue and possess the ability of scarless wound healing; they also produce the extracellular matrix in the oral connective tissues, express some cell surface proteins, and produce pro-inflammatory cytokines [33]. They are useful and interesting for the in vitro assessment of dental materials, especially for tissue engineering purposes. As far as we know, there are no studies concerning the in vitro evaluation of the biological activities against *Candida* spp. exhibited by heparin-based metal nanoparticles synthesized by photochemistry. The aim of this study was the synthesis of heparin-coated gold nanoparticles (AuHep-NPs) and heparin-coated silver nanoparticles (AgHep-NPs), their physicochemical characterization, and the assessment of their antifungal (fungistatic or fungicidal) effect on *C. albicans*, *I. orientalis*, and *C. parapsilosis*, as well as of their cytotoxicity on human HGFs, to define their potential use in the dentistry area.

## 2. Results

### 2.1. UV–Vis Spectroscopy

**Figure 1:**
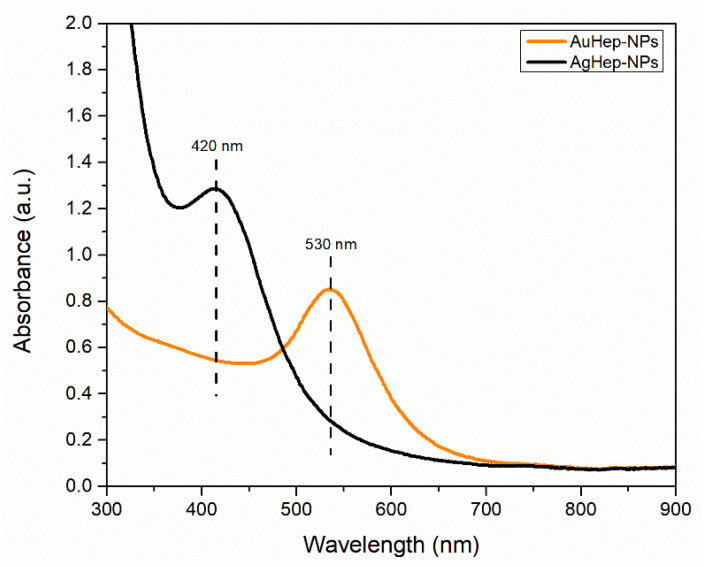
UV–Vis spectra showing plasmon resonance bands of the synthesized heparin-coated gold nanoparticles (AuHep-NPs) and heparin-coated silver nanoparticles (AgHep-NPs).

Absorption spectra were taken to determine the presence of nanoparticles and to verify that their plasmon resonance band peaks corresponded to the reported ones typical for gold and silver nanoparticles. The UV–Vis spectra in Figure 1 show that the gold nanoparticles had their maximum plasmon peak at 530 nm, whereas the maximum peak for the silver nanoparticles was at 420 nm, in the usual range for gold [34] and silver [35] spherical and pseudospherical nanoparticles, respectively.

### 2.2. Transmission Electron Microscopy

**Figure 2:**
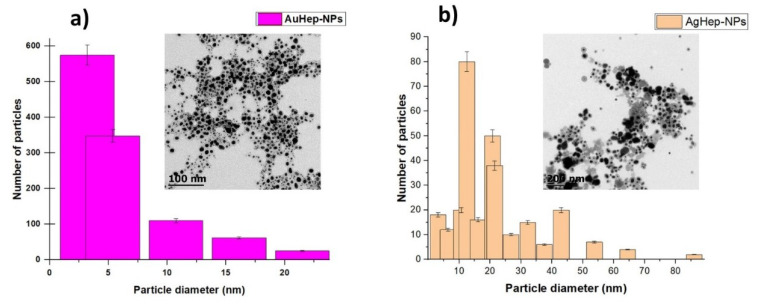
Transmission Electron Microscopy (TEM) micrographs: (**a**) AuHep-NPs and (**b**) AgHep-NPs, showing nanoparticles’ morphology as well as size.

Transmission Electron Microscopy (TEM) micrographs were used to obtain histograms showing the size distribution of the nanoparticles and to visualize their morphologies. As shown in Figure 2, the predominant diameter of the gold nanoparticles was in the range of 1.5–7.5 nm, whereas the predominant diameter of the silver nanoparticles was in the range of 10–25 nm; this means that AuHep-NPs were more monodisperse than AgHep-NPs.

### 2.3. Raman Spectroscopy

**Figure 3:**
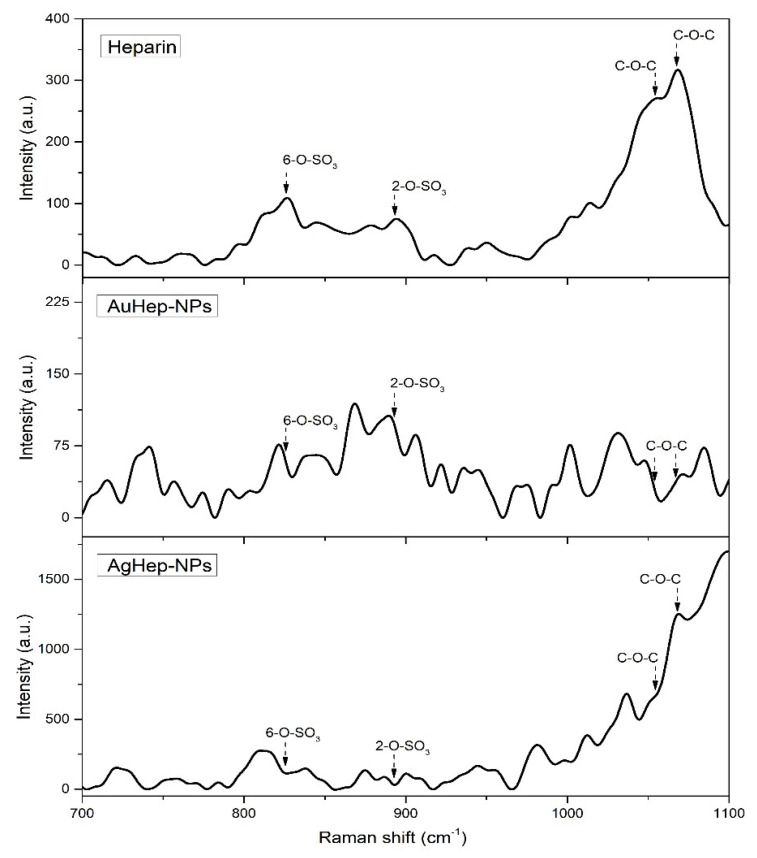
Raman spectra of heparin and those of AuHep-NPs and AgHep-NPs.

Raman spectra were used to investigate the functional groups on the capping layer present on the surface of the heparin-synthesized nanoparticles. The spectra in Figure 3 show bands corresponding to heparin in the positions 1054 cm^−1^ and 1068 cm^−1^, corresponding to the 6-O–SO_3_ and 2-O–SO_3_ vibrations, respectively. Bands at 893 cm^−1^ and 826 cm^−1^ correspond to the C–O–C vibrations [36,37,38]. In the spectrum of the gold nanoparticles, it was observed that the 6-O–SO_3_ and 2-O–SO_3_ heparin bands kept their original shape and showed a slightly weaker intensity and a red shift in comparison with the heparin spectrum. For the AgHep-NPs nanoparticles, these bands did not show shifts and were sharp. For C–O–C vibrations related to AuHep-NPs nanoparticles, the corresponding bands showed a blue shift, and their intensities were weaker in comparison to those of pure heparin. In the case of the silver NPs, the first peak of heparin at 826 cm^−1^ disappeared, and the one at 893 cm^−1^ blue-shifted and showed a stronger intensity. The enhanced intensities of the bands of heparin for the AgHep-NPs’ surface resembled a SERS (surface enhanced Raman spectroscopy) effect [36,37]. On the other hand, such intense bands were not observed in the AuHep-NPs’ spectrum; this fact is not surprising because it is known that, in general, gold nanoparticles present moderate signals [38,39]. These findings also confirmed the presence of heparin in the synthesized nanoparticles, as observed in the TEM micrographs in Figure 2.

### 2.4. Antifungal Susceptibility Test

The broth microdilution method results are presented in Table 1, with a comparison of the effects of heparin, commercial silver and gold nanoparticles, and the synthesized AgHep-NPs and AuHep-NPs. First, by comparing the performances of the synthesized heparin-based nanoparticles and heparin, it was observed that heparin did not exert any antifungal effect on the three tested *Candida* spp., which grew in all wells. This is in agreement with studies reporting the lack of antimicrobial activity in heparin [19,22]; therefore, no MFC (minimum fungicidal concentration) nor MIC (minimum inhibitory concentration) were determined. If the studies that affirm heparin possesses antimicrobial properties (either bacteriostatic or fungistatic) are taken into account, they mainly rely on factors such chemical modifications of heparin [23] and the presence of contaminants or the shortage of nutrients in the growth media [19]. In our case, heparin was not modified, and the growth media were prepared and used according to the CLSI protocol for antifungal susceptibility. Likewise, AuHep-NPs showed lack of antifungal activity. Contrary to the described heparin and AuHep-NPs behavior, the MIC values for AgHep-NPs could be acquired, hence indicating fungistatic properties.

In additin, when comparing AgHep-NPs and AuHep-NPs with their non-coated counterparts, both types of gold nanoparticles did not show any antifungical effect. Contrarily, commercial AgNPs and AgHep-NPs were found to inhibit all Candida spp., with some differences.

### 2.5. MTT Assay

**Figure 4:**
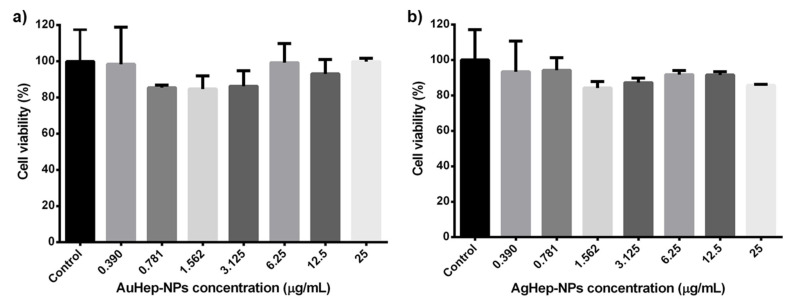
In vitro cytotoxic assay for heparin-based (**a**) AuHep-NPs and (**b**) AgHep-NPs, measuring the viability of human gingival fibroblasts (HGFs) in the presence of the nanoparticles with respect to that of HGFs in DMEM (control).

The cell viability graphs in Figure 4 show the results of the MTT assay on HGFs in contact with AuHep-NPs and AgHep-NPs after 24 h; as a control, we used HGFs in DMEM medium, which corresponded to 100% cell viability. From the bar graphs in Figure 4, it is observed that there are no significant differences between the effects of gold and silver nanoparticles compared with the control (untreated HGFs), as indicated by the *p* values (*p* = 0.40). Both nanoparticle types preserved cell viability above 80%, resulting slightly cytotoxic to a similar extent [40,41].

## 3. Discussion

In our previous study, gold nanoparticle synthesis was carried out using 366 nm black light lamps and no magnetic stirring. The previously obtained products had a plasmon resonance band with a peak at 536 nm and a band to the right at 630 nm. A full width at half medium (FWHM) was calculated using a Gaussian approximation in OriginPro 8 (OriginLab Corporation, Northampton, MA, USA) of 132.9 nm, which is higher than the value (102.7 nm) calculated for the gold nanoparticles synthesized in this work. The FWHM value is an indicator of the size dispersion of gold nanoparticles [41], and therefore, it can be concluded that changes introduced in this synthesis protocol aided in obtaining a more uniform distribution of the gold nanoparticles. Khalid et al. reported an FWHM value of 79.42 nm for silver nanoparticles synthesized with plant extracts, indicating a uniform and small-sized distribution [42] The calculated FWHM for AgHep-NPs was 72 nm, which is below this reported value, suggesting a similar nanoparticle distribution.

Giorgi-Coll et al. synthesized heparin-coated gold nanoparticles of 15 ± 1.3 nm [43], and another work by Bener et al. reported that the size nanoparticles of the same type was 42 nm [44]. In the first case, the nanoparticle size is comparable to the values obtained in the present study, while in the second case, the nanoparticle size is bigger than the largest size obtained in the present work.

There are very few reports on heparin-coated silver nanoparticles, namely, those by Kemp, who reported a nanoparticles size distribution of 11 ± 3 nm [23], and that by Huang, reporting a size distribution of 20 ± 8 nm [24]. These reports confirm a similar size distribution to the one obtained by our research group. Finally, the presence of an organic layer surrounding the nanoparticles as a result of the heparin encapsulation is according to the findings reported by Sun et al., who described heparin coating layers on the surface of the synthesized gold nanoparticles [29], as shown in Figure 3.

Kumar et al. reported MICs of 4 μg/mL and 8 μg/mL for *C. parapsilosis* and *I. orientalis*, respectively, using silver nanoparticles (pseudospherical and 13 nm in diameter) synthesized with the culture supernatant of *Pseudomonas aeruginosa* [45], evidencing a fungistatic effect similar to the one obtained in this study. However, the MIC values obtained in our study are lower than those obtained by these authors, who used AgNP in the 10–25 nm range. Kemp et al. synthesized silver DAPHP (spherical, modified heparin-based nanoparticles, 11 ± 3 nm in size), which showed bacteriostatic activity against *Staphylococcus aureus* and *Escherichia coli* within an 18 h treatment. In our study, we observed only an inhibitory effect on *C. parapsilosis* and *I. orientalis* after a 24 h exposure to AgHep-NPs. *C. albicans* was better inhibited by the commercial AgNPs than by AgHep-NPs. It has been reported that physicochemical factors such as nanoparticle size (no larger than 50 nm) and shape influence the antimicrobial activity of silver [46]. The results obtained with commercial AgNPs and AgHep-NPs agree quite well with this, but the difference in the recorded MIC values could be related to the capping agents involved, especially when referring to *C. albicans*. The commercial silver nanoparticles are not capped and are redispersed in triethyleneglycol monoethyl ether, which is a compound that has been studied for its biocidal properties on *Cladosporium resinae, Gliomastix* spp., *Candida* spp., and *P. aeruginosa* in the aircraft fuels industry. This research work proposed that their mechanism of biocidal action could be related to the damage induced on microorganisms’ cytoplasmic membrane and to osmotic effects [47]. Plus, uncapped silver nanoparticles have been reported as antimicrobial agents, but with a high toxicity level [48]. The enhanced fungistatic effect of the commercial silver nanoparticles on *I. orientalis* and *C. albicans* might be promoted in a synergistic manner by the absence of a capping agent and by traces of triethyleneglycol monoethyl ether. As for heparin, it has been reported that it enhances the formation of *C. albicans* biofilms in catheters due to heparin-binding motifs that promote cell-to-cell adhesion, resulting in the proliferation of the pathogen as a biofilm [49]. This might explain the higher MIC for AgHep-NPs against *C. albicans*. Despite the differences in MICs implying that commercial AgNPs can be much better antifungal agents, it is worth mentioning that for applications in dentistry or any other biomedical field, this is not a disadvantage because biocompatible materials are useful and promising for their potential application in clinical practice, which justifies the use of heparin for the synthesis of silver nanoparticles. If heparin alone had any antifungal properties, AuHep-NPs and AgHep-NPs would have shown it by displaying enhanced activity with respect to uncoated NPs, which was not the case for the present study.

Research works comparing AuNPs and AgNPs antifungal activity have reported that AgNPs work much better as antimicrobial agents than their gold counterparts, which is in accordance with this study [50] Furthermore, it was reported that AgNPs concentrations below 100 mg/mL result in high toxicity, due to interactions with redox enzymes, besides the destruction of enzymes in human cells [51], which might explain the observed fungistatic effect. Nevertheless, the AgNPs synthesized in this work have a therapeutic potential due to the fact that, despite their fungistatic effect and small size, they preserve cell viability, as it will be discussed later on. For AgHep-NPs, the fungistatic effect may be related to the colloidal nature of the particles, their attachment to the microorganisms’ cell membrane, or the release of silver ions (Ag^+^) [52]; further studies will have to be performed to prove these hypotheses. The lack of antifungal activity displayed by AuHep-NPs is in agreement with previous studies [53].

It has been reported that heparin derived from certain fish exhibits antimicrobial activity against bacterial and yeast species at high concentrations [54], but the exact mechanism is not clear. Also, this is not in accordance with the results observed in this study, because even at high concentrations, heparin did not exhibit any antifungal effect. On the contrary, it has been reported that heparin as a catheter locking solution provides a limited antifungal effect in the long run; therefore, it has to be combined with antibiotics to prolong this effect [22]. In any case, if heparin had an antifungal effect, it would have been observed when heparin was tested alone and it might have been enhanced when heparin was used as a capping agent for the AgHep-NPs and AuHep-NPs. It will be interesting to identify the components of heparin which might promote antifungal activity.

Nevertheless, the reported results for AgHep-NPs support the use of these NPs as additives for the improvement of denture resins [55] or as a catheter locking solution instead of heparin alone, possibly in conjunction with antimicrobial agents [56] or molecules that enhance their antifungal activity [57].

Sun et al. found that heparin-coated gold nanoparticles (54.6 ± 19.6 nm), tested in human hepatocellular liver carcinoma (hepG2) cells in the 0.1–100 mg/mL concentration range, turned out to be non-cytotoxic in a dose-dependent manner. This differs from our results: cell viability of HGFs was not dose-dependent. In addition, both our AuHep-NPs and AgHep-NPs were much smaller and resulted slightly cytotoxic. Gold nanoparticles coated with polysaccharides other than heparin did not interfere with cell growth, as shown by cell viability tests [58]. For example, Vijayakumar et al. [59] studied the viability of PC-3, MCF-7, and CHO22 cells exposed to gold nanoparticles coated with citrate, starch, and gum Arabic (20 nm). Their results showed that cell viability was preserved after 24 h, even at the highest concentration they tested, i.e., 140 μg/mL, although it was slightly lower for citrate-coated nanoparticles. The highest cell viability they obtained was with nanoparticles coated with gum Arabic, which is a natural polysaccharide. Similarly, in our case, the viability of HGFs treated with our heparin-based gold and silver nanoparticles at concentrations up to 25 μg/mL was comparable to that of the control cells (untreated HGFs), supporting the fact that capping agents have to be taken into account when synthesizing nanoparticles for biomedical purposes. Other studies of gold nanoparticles also showed a good preservation of cell viability (about 90%) [60] and significant biocompatibility [61], but the concentrations used were as high as 150 μg/mL, in contrast with the lower concentrations used in the present study.

Regarding silver nanoparticles, Inkielewicz-Stepniak et al. [62] evaluated the cell viability of human gingival fibroblasts exposed to a mixture of 2 nm commercial silver nanoparticles (US Research Nanomaterials, Houston, TX, USA) and fluoride for 24 h. They found that, when using nanoparticle in the 2.5–3.5 μg/mL concentration range, cell viability was below 50%, indicating moderate cytotoxicity. In the present study, when using concentrations of silver nanoparticles in the same range of the referred research, cell viability of human gingival fibroblasts was much higher. In another study by Niska et al. [63], it was found that the coating agent plays an important role in silver nanoparticles’ cytotoxic effects. The authors showed that treatments with lipoic acid- and polyethylene glycol-capped silver nanoparticles showed better cell viability (between 20–100%, in a dose-dependent manner) than treatments with tannic acid-capped or uncapped NPs with sizes around 10 nm. The preservation of cell viability might be related to the fact that heparin is an excellent biocompatible biomaterial, despite that fact it has been reported that it exerts some toxic effects in L929 cells, for instance, though only at really high doses [64].

The cytotoxicity results obtained in this work for AgHep-NPs in combination with a fungistatic effect allow for the possibility of developing antifungal-conjugated systems of nanoparticles for periodontitis treatment, which preserve human gingival fibroblasts viability [65]. They might also be appropriate for denture base resins [66]. Considering cell viability in the presence of AuHep-NPs, these NPs may be adequate, for example, for research related to the delivery of molecules into human gingival fibroblasts [67] and for dental imaging [68].

## 4. Materials and Methods

### 4.1. Materials

For nanoparticle synthesis, gold (III) chloride trihydrate (HAuCl_4_·3H_2_O, Aldrich, Saint Louis, MO, USA) and silver nitrate (AgNO_3_, Sigma Aldrich, Saint Louis, MO, USA) salts were used as metal precursors. Heparin sodium salt from porcine intestinal mucosa (H4784, Sigma-Aldrich, MW: 16 kDa) was used as the reducing and stabilizing agent. Benzyl alcohol (KARAL SA de CV, Leon, Guanajuato, Mexico) and deionized water were used as solvents. For the antifungal susceptibility studies, *C. albicans* (ATCC 90028), *C. parapsilosis* (ATCC 22019), and *I. orientalis* (ATCC 6258) were purchased from Microbiologics (St. Cloud, MN, USA). RPMI Medium 1640 with glutamine, without bicarbonate, with phenol red (Gibco^®^ by Life Technologies) and Sabouraud dextrose agar were used as the growth media. The controls used were heparin sodium, commercial silver nanoparticles (736481, Sigma-Aldrich, ≤50 nm particle size, 30–35 wt. % in triethylene glycol monoethyl ether), gold nanoparticles (741965, Sigma-Aldrich, citrate-stabilized, 20 nm in diameter), and Itroconazole (Sigma-Aldrich). For cytotoxicity analysis, a primary culture of HGF cells, supplemented Dulbecco’s Modified Eagle’s Medium, high-glucose growth medium (D5671, Sigma Life Science), MTT (3-(4,5-dimethylthiazol-2-yl)-2,5-diphenyltetrazolium bromide, (M2003, Sigma), and dimethyl sulfoxide (DMSO, D2650, Sigma) were used. The control used was HFGs in DMEM. Ninety-six-well plates (COSTAR 3595 from Corning Incorporated, New York, NY, USA) were utilized in both tests.

### 4.2. Nanoparticle Synthesis

The method used for the synthesis of the nanoparticles was reported by us before [26], with modifications such as the use of fungicidal lamps of 254 nm wavelength as the irradiation source and magnetic stirring of the aqueous solutions during the procedure.

#### 4.2.1. Stock Solutions Preparation

A heparin sodium solution (4 mg/mL) was prepared in benzyl alcohol/deionized water mixed at a 1:106 ratio. The precursor aqueous solutions of gold and silver were prepared in deionized water at 0.5 mM each. A heparin sodium/gold (III) chloride trihydrate solution and a heparin sodium/silver nitrate solution were prepared for gold (AuHep-NPs) and silver nanoparticles (AgHep-NPs) synthesis, respectively.

#### 4.2.2. Synthesis Procedure

Once the mixture of either aqueous chloroauric acid or silver nitrate and heparin were prepared, they were irradiated with the germicidal lamps under magnetic stirring for seven hours in separate experiments: one experiment to synthesize the gold nanoparticles and the other for the silver nanoparticles. The obtained nanoparticles were centrifuged (SIGMA 1-16K centrifuge, Germany) at 16,000 rpm for 30 min in three rounds and were finally redispersed in deionized water.

### 4.3. Nanoparticle Characterization

The obtained nanoparticles were characterized by UV–Vis spectroscopy (Thermo Scientific Multiskan GO spectrophotometer, Finland) to verify the presence of nanoparticles and that their plasmon resonance values corresponded to the typical values for gold and silver nanoparticles. To observe nanoparticles’ morphology, distribution, and size, transmission electron microscopy (JEM-1010 equipment JEOL, Peabody, MA, USA) was used along with the Digital Micrograph 3.1 software. The samples were prepared on copper grids supporting a thin film of amorphous carbon. A microbalance (Mettler Toledo XP2U, Switzerland) was used to determine the nanoparticles concentration in µg/mL. The samples were dried and weighed, and their concentrations were recorded, to be used later in the biological tests. To analyze the functional groups of heparin and the ones on the nanoparticles, Raman spectra were recorder with a Senterra Bruker Dispersive (Billerica, MA, USA) system, using the 785 nm laser line and 20× and 50× objectives at 10 mW, with integration times of 8 s and 6 s scans in the 700–1200 cm^−1^ range.

### 4.4. Antifungal Susceptibility Test

The antifungal activity assay against *Candida* spp. was carried out according to the CLSI document M27-A3-2008 [69], with slight modifications. The values of the minimum inhibitory concentration (MIC) and the minimum fungicidal concentration (MFC) in μg/mL of each type of nanoparticle synthesized against the tested *Candida* strains were determined. MIC refers to the lowest concentration of an antimicrobial agent that is bacteriostatic (inhibits the visible growth of bacteria). MFC is the minimum concentration of an antimicrobial agent required to kill a bacterium or fungus over a fixed, somewhat extended period, such as 18 h or 24 h, achieving 99.9% cell death. First, nanoparticles were dried and redispersed in the culture medium RPMI-1640 with added glucose (2%), obtaining two-fold concentrated dilutions (100 μL, starting from 25 μg/mL). Next, a standard amount of 1 × 103 colony-forming units per milliliter (CFU/mL) in 100 µL of RPMI medium were pipetted into each well of a ninety-six-well microtiter plate prepared with the fixed nanoparticle concentrations. Afterward, the ninety-six-well plates were incubated for 24 h at 37 °C, and the optical density values at 630 nm were obtained using a spectrometer (Thermo Scientific Varioskan Flash Spectral Scanning Multimode Reader, Finland). The MIC values were determined by the presence of an optically clear well in the plate. To obtain the minimum fungicidal concentration, samples of 20 µL were taken out of each well, subcultured on Sabouraud dextrose agar plates, and incubated at 37 °C for 24 h. The MFC values were recorded if no growth of yeast colonies was observed on the plate surfaces. All experiments were performed in triplicate. Commercial silver and gold nanoparticles were used to measure the effect of metallic nanoparticles at the same concentrations of heparin-coated NPs. Finally, the antifungal activity of a heparin solution (1 mg/mL) was tested.

### 4.5. MTT Assay

The MTT test was performed to evaluate the percentage of cell viability of HGFs in contact with solutions of each type of the synthesized NPs and their corresponding dilutions according to the ISO 10993-5:2009 [70]. First, human gingival fibroblasts were grown in DMEM medium supplemented with 10% FBS (Sigma) until 80% confluence was attained. Then, they were harvested using trypsin–EDTA and counted with a hemocytometer. Their cell density was adjusted by placing 1 × 10^5^ cells in each well of 96-well plates in 100 µL of DMEM medium. Afterward, the dried AuHep-NPs and AgHep-NPs were redispersed and diluted in DMEM medium in order to obtain two-fold concentrations. These dilutions were placed in aliquots of 100 µL in each well; the experiment was done in triplicate. The plates were incubated for 24 h (BINDER CB CO_2_ incubator at 37 °C, 5% CO_2_, and 95% humidity atmosphere). Following the incubation period, DMEM was removed from all wells, and 20 µL of MTT reagent (5 mg/mL) and 80 µL of fresh DMEM medium were added. This step was followed by a 7 h incubation, after which the MTT reagent was removed, and 100 µL of DMSO were poured into each well to dissolve the formazan crystals formed by the live cells.

Finally, the absorbance was recorded at a wavelength of 570 nm. Cell viability was calculated as a percentage, using Formula (1) [71], plotted, and analyzed statistically with one-way ANOVA; the statistics were analyzed using Dunnett’s multiple comparisons test and Prisma Graphpad version 6.01.

(1) $$\%Cell\;viability=\frac{Experimental\;OD_{570\;nm}}{Contro\;OD_{570\;nm}}$$

Cytotoxicity was rated based on cell viability relative to that of the controls (untreated human gingival fibroblasts) as [59,72]:

- Non-cytotoxic (>90% cell viability);
- Slightly cytotoxic (60–90% cell viability);
- Moderately cytotoxic (30–59% cell viability);
- Severely cytotoxic (≤30% cell viability).

## 5. Conclusions

The present research work showed that a photochemical UV light-based method using heparin as a reducing and a stabilizing agent yielded small-sized AuHep-NPs (1.5–7.5 nm) and AgHep-NPs (10–25 nm), as confirmed by UV–Vis and Raman spectroscopies as well as transmission electron microscopy. It was found that AgHep-NPs possessed fungistatic properties against *C. albicans*, *I. orientalis*, and *C. parapsilopis* at low concentrations, whilst AuHep-NPs did not show any antifungal activity whatsoever. Furthermore, both nanoparticle types turned out to be slightly toxic on human gingival fibroblasts at low concentrations. These results indicate the potential of these metal–heparin-based nanoparticles as candidates for some promising applications in biomedicine and to the greatest extent, in dentistry.

## Figures and Tables

**Table 1:** MIC and MFC values of heparin-coated (AuHep-NPs and AgHep-NPs) and non-coated gold and silver NPs against *Candida* species, tested along with Itroconazole used as a control.

Tested Agent	*Candida parapsilosis* MIC/MFC (μg/mL)	*Issatchenkia orientalis* MIC/MFC (μg/mL)	*Candida albicans* MIC/MFC (μg/mL)
Itraconazole	0.250/0.250	0.50/0.50	>16 (resistant)
AgNPs (non-coated)	3.125/12.5	3.125/12.5	6.25/>25
AgHep-NPs	3.125/>25	6.250/>25	25/>25
AuNPs (non-coated)	>25	>25	>25
AuHep-NPs	>25	>25	>25
Heparin solution	>1000/>1000	>1000/>1000	>1000/>1000
